# Evaluating malocclusion patterns in children with autism spectrum disorder using the index of complexity, outcome and need: a cross-sectional study

**DOI:** 10.1186/s12903-024-04524-y

**Published:** 2024-07-04

**Authors:** Fahimeh Farzanegan, Sahar Ahmadi Shadmehri, Zahra Shooshtari, Amir Reza Hamidi, Arsalan Shahri

**Affiliations:** 1 Department of Orthodontics, Dental Materials Research Center, Mashhad Dental School, Mashhad University of Medical Sciences, Mashhad, Iran; 2 Student Research Committee, School of Dentistry, Mashhad University of Medical Sciences, Mashhad, Iran; 3 Research Assistant, Dental Research Center, Mashhad Dental School, Mashhad University of Medical Sciences, Mashhad, Iran; 4 Dental Materials Research Center, Mashhad Dental School, Mashhad University of Medical Sciences, Mashhad, Iran

**Keywords:** Autism spectrum disorder, Dental care for children, Malocclusion, Orthodontics, Prevalence

## Abstract

**Background:**

The purpose of this study was to evaluate the complexity of malocclusion and existing patterns in children with autism spectrum disorders (ASD) using the index of complexity, outcome and need (ICON).

**Methods:**

This cross-sectional study included children diagnosed with ASD, aged 9–15 years. A group of healthy children with the same demographic characteristics was randomly selected as the control group. Malocclusion was assessed according to ICON scoring protocol. The following parameters were recorded: dental aesthetics, upper arch crowding/spacing, presence of crossbite, anterior-vertical relationship (open and deep bite) and buccal segment anterior-posterior relationship. Finally, an overall ICON score was derived and reported for each patient. Descriptive analysis was performed for all investigated variables. Significance level was set at *p* < 0.05.

**Results:**

A total of 324 children, divided into ASD (162) and control (162) groups, comprised the study population. Our results demonstrated that the average overall ICON score was significantly higher in the ASD group compared to the control group (38.77 vs. 27.43, *p* < 0.001). ASD children also obtained significantly higher scores regarding the dental aesthetics component (3.84 vs 2.78, *p* < 0.001). Study groups were significantly different in terms of the prevalence of incisor overbite and open bite (*p* = 0.002 and *p* < 0.001, respectively). Patients in the ASD group showed a higher prevalence of Class II and Class III malocclusions (*p* < 0.001).

**Conclusion:**

ASD children obtained significantly higher overall ICON scores, indicating more complex and severe malocclusions. These children also exhibited a greater tendency towards Class II and III malocclusions.

## Background

Autism is referred to as a spectrum of neurodevelopmental disorders characterized by impaired social interactions, communication skills, and repetitive, restricted, and stereotypical behavioral patterns [1, 2]. The prevalence of autism disorder has drastically increased over the past decades [2]. According to 2022 data, approximately 1 in every 100 children receives a medical diagnosis of autism spectrum disorder (ASD) worldwide [3]. The latest national reports indicate a prevalence of 19 per 1000 of ASD among elementary school students in Iran [4].

ASD is deemed to be of multifactorial etiology. Although both environmental and genetic factors can contribute to the development of autism, a stronger correlation between autism and environmental factors has been proposed. Advanced parental age, maternal stress, and intrauterine exposure to teratogenic drugs can increase the risk to autism development in the newborn child [5]. ASD encompasses a broad spectrum of mental health conditions, sensory impairments, and physical disabilities. Minor malformations such as small feet, large hands, and posterior rotation of the external ear have also been observed in this population [6]. Due to its proven association with the central nervous system, autism has also been linked with a higher prevalence of morphologic abnormalities such as craniofacial deformities [7]. Similar to other conditions affecting craniofacial development, as described previously, these abnormalities can have significant implications for health and development [8].

Population-based studies have identified a marked increase in the prevalence of autism in recent decades. This disorder imposes a significant medical, psychological, social, and financial burden on ASD individuals, their families, and society as a whole [9–11]. These children are less likely to have adequate access to dental health care services, especially orthodontic care, making them more prone to developing complex malocclusions in later stages. Thus, this population’s oral health and orthodontic needs constitute significant concerns and deserve particular attention. Vigorous screening and early identification of malocclusion are necessary for establishing more preventive strategies and can also improve service planning for these patients.

Angle’s classification of malocclusion and the index of complexity, outcome and need (ICON) are typically used to assess the prevalence of malocclusion in different populations. ICON was first introduced by Daniels et al. in 2000 [12] and is used to evaluate treatment need, complexity, treatment improvement, and outcome in orthodontic patients. This index was designed and generated by integrating the opinions of 97 orthodontics and has been purported to provide accurate and reliable results when used in different ethnic groups [12, 13].

The relevant literature confirms a higher prevalence of malocclusion in those with intellectual disabilities or the mental compromise. Malocclusion, requiring treatment, has been recognized in 74% of patients with mental retardation or developmental disabilities [14]. Numerous studies have evaluated the prevalence of malocclusion and different malocclusion traits in patients with various physical or mental disabilities. The literature suggests a higher prevalence of malocclusion in children with special health care needs, compared to healthy individuals [15–22]. The prevalence of malocclusion has gained increasing interest over the years and has already been evaluated in various ethnic groups. However, our literature review reveals a paucity of studies investigating this matter in the Iranian population. Hence, the purpose of the present study was to assess the prevalence of malocclusion and identify the different malocclusion traits in ASD children residing in Mashhad, Iran, using ICON.

## Methods

### Study design

The present study was conducted from September 2021 to January 2022 in Mashhad, Iran. Study groups consisted of children with ASD and healthy children. The protocol was approved by the Research and Ethics Committee of Mashhad University of Medical Sciences (IR.MUMS.DENTISTRY.REC.1398.042).

A list of registered rehabilitation centers for autistic children was obtained from the Ministry of Education and also the Government Social Welfare Department in Mashhad, Iran. This list included a total of 11 institutions, which we contacted, and the study purpose was explained; they were then invited to take part in this study. Ten centers agreed to the study protocol and granted permission, while one center refused to participate in this study. Written informed consent was sought from the children’s parents/ primary-care givers prior to enrollment.

Inclusion criteria were children with a confirmed medical diagnosis of ASD within the age range of 9–15 years old for the ASD group, presence at the mentioned welfare institutions, and healthy children with the same demographic characteristics for the non-ASD group, recruited from mainstream elementary schools in different districts of the city. Exclusion criteria were children with any other syndromes or disorders aside from ASD, those with craniofacial deformities, children with a history of orthodontic therapy or those currently undergoing treatment, and those with severe conditions of autism that could not cooperate.

### Data collection

A trained and calibrated dental student was assigned to carry out the intraoral examinations. Before commencing the actual clinical examinations, 10 patients; aged between 9 and 15 years old, who attended the Orthodontic Department of Mashhad School of Dentistry, were randomly selected. Each individual was separately examined twice, once by an experienced orthodontic specialist and once by the dental student. Intraoral examinations were completed in accordance with ICON criteria. Intraexaminer reliability was assessed using intraclass correlation coefficient (ICC). An ICC value of 0.96, indicating excellent reliability, was obtained. Therefore the dental student was qualified to perform the dental examinations on all the study participants and collect the required data.

Examinations took place in rehabilitation centers and preschools and were performed by one of the authors (SA). During the examination procedure, the children were to sit in a chair in an upright position while their head was in a natural position. An intraoral mirror, gauze and WHO probe were used for clinical examinations. A corresponding chart was designed for each child, in which their demographic information as well as parameters related to malocclusion were recorded. Malocclusion was assessed by the index of complexity, outcome and need (ICON). Five parameters such as dental aesthetics, upper arch crowding/spacing, the presence of crossbite, anterior-vertical relationship (open and deep bite) and buccal segment anterior-posterior relationship were assessed and scored in this index [Table 1]. A final score is derived from multiplying the raw scores by their respective weights and adding them together. An overall weighted score of 31 or less is interpreted as “acceptable an no treatment needed” and a value of 43 or greater is considered as “treatment needed” and a difficult complexity. The utilized occlusal trait scoring protocol is described in the appendix.

**Table 1:** Protocol for occlusal trait scoring

	SCORE	0	1	2	3	4	5
Aesthetic	1–10 as judged using SCAN						
Upper arch crowding	Score only the highest trait either spacing or crowding	< 2 mm	2.1–5 mm	5.1–9 mm	9.1–13 mm	13.1–17 mm	> 17 mm or impacted teeth
Upper spacing		Up to 2 mm	2.1–5 mm	5.1–9 mm	> 9 mm		
Crossbite	Transverse relationship of cusp to cusp or worse	No crossbite	Crossbite present				
Incisor open bite	Score only the highest trait either open bite or overbite	Complete bite	< 1 mm	1.1–2 mm	2.1–4 mm	> 4 mm	
Incisor overbite	Lower incisor coverage	Up to 1/3 tooth	1/3 − 2/3 coverage	2/3 up to full coverage	Fully covered		
Buccal segment antero-posterior	Left and right added together	Cusp to embrasure relationship only. Class I, II or III	Any cusp relation up to but not including cusp to cusp	Cusp to cusp relationship			

### Statistical analysis and sample size calculation

Data were subjected to statistical analysis using SPSS version 20.0 (SPSS Inc., Chicago, IL, USA). Descriptive analysis was performed for all investigated variables. The chi-square test, Mann-Whitney-U test and Kendall’s tau-b were employed for statistical analysis. A *p*-value of less than 0.05 was considered statistically significant. Sample size calculation was accomplished using the reported prevalence of malocclusion for children with and without ASD (58.6% and 35.6%, respectively), derived from previously published data [21]. Considering a type I error of 5% and a type II error of 20%, a minimum sample size of 72 subjects was estimated for each group.

## Results

A total of 324 children, divided into ASD (162) and control (162) groups, comprised the study population. Participants had an average age of 11.87 ± 1.95 years and an age range of 9–15 years. Gender distribution frequency consisted of 272 males (84%) and 52 females (16%). Study groups were exactly the same in terms of mean age (11.87 ± 1.95 years) and gender distribution (136 males and 26 females, per group).

The mean final ICON score was calculated to be 38.77 and 27.43 in the ASD and control group, respectively. This difference was statistically significant (*p* < 0.001). The mean obtained dental aesthetic score was also significantly higher in the ASD group compared to the control group, 3.84 versus 2.78. This difference was again considered statistically significant (*p* < 0.001). Table 2 illustrates the ICON and dental aesthetic values in respect to the study groups. It is noteworthy that according to Shapiro-Wilk analysis these two variables displayed non-normal distribution, therefore non-parametric tests were employed.

**Table 2:** Final ICON score and dental aesthetic score distribution among the two groups

Variable	Group	Number	Mean	Standard deviation	Minimum	Maximum	Interquartile range (Median)	Mann-Whitney U
Final score	ASD	162	77/38	091/19	7	87	(23)5/36	Z = 76/5 *P* < 001/0
	Control	162	43/27	110/16	7	82	(21)5/24	
Dental aesthetics	ASD	162	84/3	158/2	1	9	(3)0/3	Z = 87/4 *P* < 001/0
	Control	162	78/2	719/1	1	9	(3)0/2	

Regarding the maxillary arch, intraoral examination revealed that upper arch crowding and spacing were identified in 205 (63.3%) and 119 (36.7%) of the patients, respectively. Among the 205 participants with upper arch crowding, 104 of them were in the ASD group and the remaining 101 patients were part of the control group. In both study groups, the highest prevalence of maxillary arch crowding was related to a crowding of 2 mm or less, while space deficiencies between 9.1 and 13 mm were the least frequent. Overall, no statistically significant differences were found among the distribution of upper arch crowding values (*p* = 0.118). A total of 58 ASD patients and 61 patients from the control group, proved to have upper arch spacing. Concerning the amount of dental spacing in the maxilla, the majority of patients in both groups fell within the range of 2.1 to 5 mm of spacing in the upper arch. Among participants with upper arch spacing; the least frequent recorded spacing values in the ASD group were within the range of 5.1–9 mm, with only one patient. In the control group, whereas, the lowest frequency was related to an upper arch spacing greater than 9 mm, without any recorded patients. A statistically significant difference was identified between the two study groups, in terms of this variable (*p* = 0.041). Tables 3 and 4 demonstrate these findings in greater detail.

**Table 3:** Upper arch crowding distribution among the two groups

Group		Upper arch crowding				Total
		< 2 mm	2.1–5 mm	5.1–9 mm	9.1–13 mm	
ASD	Number	67	30	4	3	104
	Percentage	64.4%	28.8%	3.8%	2.9%	100%
Control	Number	76	18	5	2	101
	Percentage	75.2%	17.8%	5.0%	2.0%	100%
Total	Number	143	48	9	5	205
	Percentage	69.8%	23.4%	4.4%	2.4%	100%
Kendall’s tau-b		Τ_b_ = 11/0 *p* = 118/0				

**Table 4:** Upper arch spacing distribution among the two groups

Group		Upper arch spacing				Total
		< 2 mm	2.1–5 mm	5.1–9 mm	> 9 mm	
ASD	Number	24	31	1	2	58
	Percentage	41.4%	53.4%	1.7%	3.4%	100%
Control	Number	17	34	10	0	61
	Percentage	27.9%	55.7%	16.4%	0%	100%
Total	Number	41	65	11	2	119
	Percentage	34.5%	54.6%	9.2%	1.7%	100%
Kendall’s tau-b		Τ_b_ = 18/0 *p* = 041/0				

Posterior crossbite was observed in a total of 70 (21.6%) participants, 41 (25.3%) of whom were diagnosed with ASD and 29 (17.9%) healthy children. Although the prevalence of posterior crossbite was slightly higher in the ASD group, Chi-square test revealed this difference to be statistically insignificant (*p* = 0.105).

As displayed in Table 5, among the 254 (78.4%) patients categorized as having incisor overbite, most cases had 1/3 to 2/3 incisor coverage and only 10 cases scored as fully covered. The ASD and control groups were significantly different regarding the prevalence of incisor overbite (*p* = 0.002). On the other hand, incisor open bite was only observed in 70 (21.6%) patients, 39 with ASD and 31 healthy. The highest frequency regarding this variable was related to an edge to edge incisor relationship, while an interincisal distance greater than 4 mm was the least common in both study groups. The prevalence of incisor open bite was found to be significantly different among ASD and non-ASD children (*p* < 0.001). Table 6 also presents these results.

**Table 5:** Incisor over bite distribution among the two groups

Group		Incisor over bite				Total
		< 3/1 coverage	3/2–3/1 coverage	3/2 to fully covered	Fully covered	
ASD	Number	28	50	37	8	123
	Percentage	22.8%	40.7%	30.1%	6.5%	100%
Control	Number	32	84	13	2	131
	Percentage	24.4%	64.1%	9.9%	1.5%	100%
Total	Number	60	134	50	10	254
	Percentage	23.6%	52.8%	19.7%	3.9%	100%
Kendall’s tau-b		Τ_b_ = 19/0 *p* = 002/0				

**Table 6:** Incisor open bite distribution among the two groups

Group		Incisor open bite					Total
		Edge to edge	< 1 mm	1.1–2 mm	2.1–4 mm	> 4 mm	
ASD	Number	12	6	11	9	1	39
	Percentage	30.8%	15.4%	28.2%	23.1%	2.6%	100%
Control	Number	27	2	1	1	0	31
	Percentage	87.1%	6.5%	3.2%	3.2%	0%	100%
Total	Number	39	8	12	10	1	70
	Percentage	55.7%	11.4%	17.1%	14.3%	1.4%	100%
Kendall’s tau-b		Τ_b_ = 52/0 *p* < 001/0					

Buccal segment anterior-posterior cuspal relationship was assessed in all consecutive patients. Class I molar relationship was registered for the majority of healthy children (63.6%). As for ASD patients, the most common molar relationships were found to be Class I and III, followed by Class II and cusp to cusp relationship, in descending order of frequency. As shown in Table 7, Chi-square test indicates that both groups displayed statistically significant differences regarding the distribution of different buccal segment interdigitation traits (*p* < 0.001).

**Table 7:** Buccal segment anterior-posterior relationship distribution among the two groups

Group		Buccal segment anterior-posterior relationship				Total
		Class I	Class II	Class III	cusp to cusp	
ASD	Number	46	30	46	40	162
	Percentage	28.4%	18.5%	28.4%	24.7%	100%
Control	Number	103	9	21	29	162
	Percentage	63.6%	5.6%	13.0%	17.9%	100%
Total	Number	149	39	67	69	324
	Percentage	46.0%	12.0%	20.7%	21.3%	100%
Chi-square test		X^2^ = 20/44 *p* < 001/0				

## Discussion

This cross-sectional study assessed the malocclusion status and patterns in a sample of ASD children, residing in Mashhad, Iran. The authors also evaluated their need for orthodontic treatment according to obtained ICON scores. A significantly higher final ICON score was documented for these patients compared to their healthy counterparts. This suggests the presence of more severe and complex malocclusions in ASD children. Furthermore, the established results also revealed a greater tendency to certain malocclusion traits such as Class II and III malocclusions and cusp to cusp buccal segment anterior-posterior relationship. On the other hand, upper arch spacing was significantly less frequent in ASD patients. Although ASD patients displayed a higher rate of posterior cross-bite and upper arch crowding, this difference was not proven to be statistically significant.

ICON was incorporated to assess the need for treatment and treatment complexity in our study population. Previous studies confirm this index to be both valid and reliable for orthodontic assessment in the Iranian population [24]. This index offers simple practical application without requiring any special equipment except for a dental millimeter ruler and table of aesthetic scores. The ICON has relatively high accuracy even when examinations are conducted by someone who is not an orthodontic specialist [23].

Depending on the severity of the physical, mental and social deficits co-occurring with autism; these patients’ psychological well-being may be compromised. Multiple medical, behavioral and morphological infirmities are also observed in children suffering from this disorder, which may be related to genetic factors or due to their autistic behavioral patterns. These patients may also struggle with maxillofacial deformities and malocclusion which can greatly affect one’s quality of life. On the other hand, the majority of autistic children do not receive preventive orthodontic therapy, this may be rationalized by the fact that oral health care needs are often overshadowed by much more urgent concerns and are not considered a priority. Dentists may also be reluctant in providing dental care for these patients with special needs, since this task is usually considered challenging. Hence, malocclusion may persist till adulthood, when treatment outcomes are less satisfactory [16–18, 21]. In this context, evaluating the prevalence of malocclusion in these patients is prudent and can foster opportune and appropriate orthodontic management as well as uncovering the underlying causes.

The prevalence of malocclusion was evaluated and compared in individuals with and without intellectual disabilities, through a study conducted by Cabrita et al. [17]. Disabled patients exhibited a higher prevalence of malocclusion compared to healthy individuals. This was similar to the findings of the present study. On the contrary, patients with special needs showed a lower prevalence of maxillary crowding; whereas the exact opposite was noticed in our study. This inconsistency may be attributable to a number of causes; Cabrita et al. used a different index for malocclusion assessment, dental crowding was only evaluated in the anterior segment and patients with other intellectual disabilities other than autism were also included in the study. Cabrita et al. reported a 24.07% prevalence of anterior open bite in autistic children; this value was relatively close to the 29.3% prevalence established in our study.

Vittek et al. analyzed the malocclusion traits in individuals with mental retardation and developmental disabilities [16]. Out of the 485 consecutive patients, 26 were diagnosed with ASD. Posterior crossbite was registered for 25% of ASD patients, which is very similar to the 25.3% prevalence reported in the present study. The prevalence rate of other malocclusion traits reported by Vittek et al. were not similar to the findings of this study which may be due to their relatively small sample size of only 26 ASD patients.

In 2017 Fontaine-Sylvester et al. conducted a cross-sectional study on a total of 200 Canadian children, 99 of whom diagnosed with ASD [21]. This study also established that ASD children exhibited a significantly higher prevalence rate of malocclusion compared to healthy children. This was in accordance with the results of the current study. A relatively lower frequency rate of anterior open bite and overbite was registered for ASD children, in Fontaine-Sylvester et al. study compared to our study (11% versus 24.07% and 23% versus 58.64%, respectively). The reason behind these differences may be because that unlike our study, the cited authors decided not to include an edge to edge incisor relationship in the incisor open bite category; and incisor overbite was confined to incisor coverage of 2/3 and greater.

A study by Udhya et al. reviewed the dental literature pertaining to the oral health status and dental management of patients with autism disorder [20]. The selected and existing studies, published prior to 2014 (from 1969 to 2014), were also indicative of a greater prevalence of malocclusion in autistic children when compared to the healthy population.

Vellappally et al. examined a total of 243 adolescents with various mental disabilities, in aim of investigating the prevalence of malocclusion and its association with dental caries in this Indian population [25]. The established results displayed a significantly higher prevalence rate of malocclusion compared to healthy individuals. However, regarding autistic children who only comprised 14 subjects, the reported prevalence rate of maxillary crowding and incisor open bite were reported to be 50% and 14.4%, respectively. These values were different from those found in our study population. This again can possibly be due to the low number of consecutive autistic children analyzed in Vellappally et al. study.

Muppa et al. also assessed the malocclusion trends in special needs children and a 20% frequency rate for incisor open bite in ASD children was established, which is relatively similar to the 24% prevalence rate reported in our ASD study sample [18].

According to the results stated by Luppanapornlarp et al., although the overall prevalence of malocclusion was not significantly different between children with and without ASD; a greater tendency to upper arch spacing, incisor open bite and Class II malocclusion was noticed in these individuals [26].

Previous studies, such as studies conducted by Vittek et al., Cabrita et al., Fontaine-Sylvestre et al., also demonstrate a higher prevalence of class II and III malocclusion in autistic children [16, 17, 21]. This corroborates the findings of the present study.

Within its limitations, this study highlights the malocclusion traits and patterns in ASD children in addition to comparing with that of healthy individuals. There are two notable limitations to consider: Firstly, considering its cross-sectional nature, it was not feasible to determine the etiologic and contributing factors to malocclusion in these patients with special needs. Secondly, the study was conducted exclusively in governmental organizations, thus excluding data from private facilities. This is an area for particular attention and merits extensive research. Further well-designed studies are required in order to draw definite conclusions and also substantiate the findings of the present study.

## Conclusion

According to the established results, malocclusion was found to be significantly more prevalent among the ASD population and ASD children obtained significantly higher overall ICON scores. Class II and III malocclusions were significantly more common in ASD children. Regarding excessive overbite, increased open bite and buccal segment anterior-posterior relationship, in each malocclusion trait, higher scores were significantly more frequent in ASD children compared to healthy individuals. Therefore, these patients usually have more complex orthodontic treatment needs and deserve meticulous planning in order to optimize orthodontic management and shift to more non-invasive interceptive treatments for this population.

## Data Availability

The data of the current study are available from the corresponding author on reasonable request.
